# The use of text mining to detect key shifts in Japanese first-year medical student professional identity formation through early exposure to non-healthcare hospital staff

**DOI:** 10.1186/s12909-021-02818-1

**Published:** 2021-07-20

**Authors:** Yayoi Shikama, Yasuko Chiba, Megumi Yasuda, Maham Stanyon, Koji Otani

**Affiliations:** Center for Medical Education and Career Development, Fukushima Medical University, 1 Hikarigaoka, Fukushima, 960-1295 Japan

**Keywords:** Professional identity formation, Role modelling, Text mining, Altruism, Socialization

## Abstract

**Background:**

Professional identity formation is nurtured through socialization, driven by interaction with role models, and supported through early clinical exposure (ECE) programmes. Non-healthcare professionals form part of the hospital community but are external to the culture of medicine, with their potential as role models unexplored. We employed text mining of student reflective assignments to explore the impact of socialization with non-healthcare professionals during ECE.

**Methods:**

Assignments from 259 first-year medical students at Fukushima Medical University, Japan, underwent hierarchical cluster analysis. Interrelationships between the most-frequently-occurring words were analysed to create coding rules, which were applied to elucidate underlying themes.

**Results:**

A shift in terms describing professional characteristics was detected, from “knowledge/skill” towards “pride [in one’s work]” and “responsibility”. Seven themes emerged: *contribution of non-healthcare professionals, diversity of occupation, pride, responsibility, teamwork, patient care and gratitude*. Students mentioning ‘*contribution of non-healthcare professionals*’ spoke of altruistic dedication and strong sense of purpose. These students expressed gratitude towards non-healthcare professionals for supporting clinical work, from a doctor’s perspective.

**Conclusion:**

Socialization with non-healthcare professionals provides important insights into the hospital working environment and cultural working norms. Through role modelling altruism and responsibility, non-healthcare professionals positively influenced student professional identity formation, promoting self-conceptualisation as a doctor.

## Introduction

If according to the well-known African proverb it takes a village to raise a child, then by this logic it should take a hospital to raise a doctor. Professional identity formation, identified as the desired outcome of teaching professionalism [1], is the process medical students undergo in their transformation from layperson to physician. Crucial to this journey is socialization into the role with increasing participation in the community, where interaction with community members drives identity development [2]. Through exposure to the beliefs, attitudes and behaviours of those encountered, identities are deconstructed and re-negotiated through conscious reflection and unconscious acquisition [3]. Role models, defined as “*individuals admired for their ways of being and acting as professionals”,* are central to professional role acquisition [4]; however, uncritical imitation of physician role models can perpetuate undesirable practice including doctor-centred practice and the reinforcement of discriminatory stereotypes [5]. Whilst it is recognised that experiences with non-clinicians, including patients and the lay public, have a role in professional identity formation [3], the focus has been on physician role models. Non-healthcare professionals working in the hospital environment occupy a unique social position; by belonging to the same institution and sharing the same professional work space they form part of the hospital c*onstellation of practice* [6], but remain external to the culture of medicine. As the expression of professionalism includes non-clinical attributes such as altruism, integrity, responsibility, respect and self-awareness [7], it would be presumptuous to assume such virtues could only be learnt from healthcare professionals; however, the potential of non-healthcare professionals to contribute to medical student professional identity development, remains unexplored to date.

Alongside role model exposure, the importance of learning environment in facilitating professional role acquisition in medical students is recognised [3, 8]. It is also acknowledged that humanistic attitudes are more effectively nurtured through experience rather than classroom lectures [9, 10]. Early clinical exposure (ECE), expressed as “*authentic human contact in a social or clinical context*”, embodies these principles and marks the opportunity for early-year students to experience the lived impact of physician professional values, from the perspective of an apprentice [11]. A common objective of ECE programmes therefore, is to help students assimilate the health professional role and acclimatise to the context in which healthcare is conducted [12, 13]. Reported outcomes show a positive impact on professional identity formation [14], increased student self-awareness [12] and emotional maturity [15]. However, to become a functioning member of the hospital community, students must also acquire knowledge of working culture within the hospital and how this supports the delivery of healthcare; such is the gap that ECE is needed to bridge.

At Fukushima Medical University, Japan, most first year students are between 18 and 19-years-old, having entered medical school directly from high school or after attending an additional year-long preparatory course. Most have little experience or knowledge of how to fulfil their role and responsibilities as employed members of Japanese society. This is compounded by a lay perspective of the hospital environment, where the work of non-healthcare professionals may be unseen or eclipsed. Through the inclusion of a ‘*behind-the-scenes’* component to our ECE programme, promoting socialization with non-healthcare professionals, we sought to increase student understanding of the role of non-healthcare professionals; specifically, how their work supports that of clinical professionals and to expose students to Japanese working-culture norms, as part of acquiring a meaningful understanding of the hospital as a workplace. In this study we used text mining to analyse student reflective assignments on the experience, to explore the impact of socialization with non-healthcare professionals during ECE on student professional identity.

## Methods

### ECE course and assignments

ECE is a compulsory course for first-year medical students at Fukushima Medical University. Following a half-day orientation, students spend 1 day touring key hospital departments and 1 day shadowing ward staff. In this study, students experienced 4 clinical and 5 non-clinical departments, where they encountered healthcare staff (doctors, nurses, pharmacists, and professions allied to healthcare); and 4 departments (medical records, the medical supply centre, boiler room and the central maintenance room) where they interacted with non-healthcare staff. Students experienced all departments during the hospital tour but were allocated a single clinical department for shadowing. Students conducted the placement in groups of 12 and varied in the order in which they completed the placement. All staff in the departments involved were requested to explain the purpose of their work and daily duties.

During the placement, students undertook a series of semi-structured reflective tasks that were later graded by faculty as part of student in-course assessment. The first task, completed after orientation, was to list the occupations needed to run a 700-bed hospital (the size of our university hospital) and summarise the key characteristics of professionals working in the hospital in two sentences totalling three lines. Students were then asked to repeat this summary after the hospital experience and again after the ward shadowing. Finally, after the placement, students were asked to write a reflection on their experiences in no more than 18 lines of text. Key characteristic summaries and reflections were collected from a total of 259 students between July 2017 and July 2018.

### Text mining and statistical analysis

Text mining encompasses a number of techniques, where algorithms are applied to extract patterns from unstructured information, allowing subsequent modelling, deeper analysis and knowledge discovery [16]. In qualitative research, text mining increases the reliability and validity of coding [17], resulting in increased rigour when used in conjunction with content analysis by human coders [18]. KH Coder 2.0 (Higuchi, Ritsumeikan University, Japan), uses the ChaSen Morphological Analyzer to combine natural language processing techniques with R statistical software and supports Japanese character input [19, 20]. Precedence for its use in the analysis of Japanese medical student reflective essays has recently been established [21]. We used KH coder 2.0 to perform a hierarchical cluster analysis, extracting the top 10% most frequently occurring words from the student statements about professional characteristics of hospital workers at all three timepoints. We optimised retrieval and interpretation through checking word usage via the KWIC concordance function. Co-occurrence, the strength of association between extracted words was calculated via the Jaccard coefficient. Significance was assessed through the application of a chi-squared test (KH coder), where α = 0.05, allowing us to track student ideas regarding professional characteristics as they evolved during the placement.

To explore the effect of the hospital tour component on students, the student reflections underwent the same extraction and hierarchical cluster analysis process, this time extracting the top 50 most commonly occurring words. Grammatical particles used in Japanese to denote the relationship between words in sentences were excluded. The resulting associations were visually represented on a co-occurrence network map (Fig. 2), coded and categorised by three authors according to the underlying themes. The reflections were then read in their entirety and the coding rules identifying the key themes applied, with the results discussed to consensus. For ease of analysis, the essays were divided into 2 groups according to which departments made the strongest impact on the students; group A, made up of students who in their own words were most affected by non-healthcare professionals and group B, which consisted of students most affected by socialization with healthcare professionals. Fisher’s exact test (IBM SPSS ver.25) was applied to (i) explore the interrelationship of the themes extracted and (ii) elaborate on the relationships between the themes and the interactions which had the greatest impact on students.

All data was transcribed and analysed in Japanese with translation into English for this report. Ethics approval was obtained from the Fukushima Medical University Ethics Committee (approval number 2019–070) and participants freely gave informed consent. All methods were carried out in accordance with relevant guidelines and regulations.

## Results

### Knowledge of hospital job type

All 259 students completed this task. Table 1 shows the job types separated into healthcare and non-healthcare professional roles. Amongst the healthcare professionals listed, doctors and nurses featured on 96.8 and 98.0% of lists respectively. Allied-health professionals were named on 64.0% of lists and pharmacists were mentioned on 46.2%. Of the non-healthcare professionals mentioned, those in positions interacting directly with the general public were most frequently listed (accounting and reception staff 71.5%, cleaners 65.2%, cooks 23.7% and security officers 21.7%); however, those with less visible *‘behind the scenes’* roles, such as maintenance technicians, medical supply managers and medical record keepers were mentioned by 5.5, 2.8 and 0.8% of students, respectively.

**Table 1:** A breakdown of the job categories listed by students after orientation

	Job categories	Percentage of students listing the profession
Healthcare professionals	Nurse	98,0
	Medical doctor	96.8
	Allied healthcare professionals^a^	64.0
	Pharmacist	46.2
Non-healthcare professionals	Office workers	71.5
	Cleaner	65.2
	Cook	23.7
	Security officer	21.7
	Maintenance technicians	5.5
	Management consultant	2.8
	Medical supply manager	2.8
	Medical records keeper	0.8
	Lawyer	0.8
	Helicopter pilot	0.8

^a^Allied health professionals consisted of physiotherapists, occupational therapists, speech and language therapists, dieticians, counsellors, healthcare assistants, radiographers and laboratory technicians

### Student perceptions of key characteristics associated with hospital professionals

Two hundred fifty-nine statements describing key characteristics of hospital professionals were submitted after orientation, 181 after the hospital tour and 178 after ward shadowing; 161 students submitted statements at all three time points. KH coder detected a total of 813 sentences comprising 377, 225 and 211 sentences at each time point respectively, made up of 1298 unique terms. After orientation but before either the hospital tour or ward shadowing, seven words emerged in more than 10% of sentences:

1. self(自分): 37.9%
2. job(仕事): 28.9%
3. knowledge(知識):18.6%
4. responsibility(責任):18.3%
5. specialty(専門): 13.5%
6. person(人): 12.2%
7. skill(技術): 11.7%

According to the KH coder KWIC concordance function, ‘self’ was connected to the Japanese particle ‘の’, followed by ‘specialty’ or ‘job’. We interpreted this as ‘own specialty/job’, which was in turn strongly associated with ‘knowledge’, ‘skill’, or ‘responsibility’ as a modifier. ‘skill’ was used interchangeably with ‘knowledge’ therefore ‘knowledge/skill’ and ‘responsibility’ were extracted. From the statements made after the hospital tour, six words were enumerated:

1. self: 48.9%
2. job: 48.9%
3. person: 30.2%
4. pride(誇り): 21.0%
5. responsibility: 19.6%
6. patient(患者): 17.8%

Amongst these, ‘self’ and ‘job’ were connected and interpreted as ‘own job’, linked with ‘pride’ or ‘responsibility’, as in ‘having pride in their job’ or ‘being diligent in their responsibilities’. ‘patient’ was used in phrases such as ‘medical care for patients’ or ‘thinking about patients’ and translated as the singular ‘patient’ or plural ‘patients’ depending on the context as these are not distinguished in Japanese. ‘pride’, ‘responsibility’, and ‘patient’ were accepted as the most frequently used words associated with characteristics of Japanese professionals after the hospital tour. Lastly, after the ward shadowing, six words were extracted:

1. patient: 39.3%
2. knowledge: 14.7%
3. medical care(医療): 11.8%
4. self: 33.6%
5. person: 29.4%
6. job: 26.1%

**Fig. 1:**
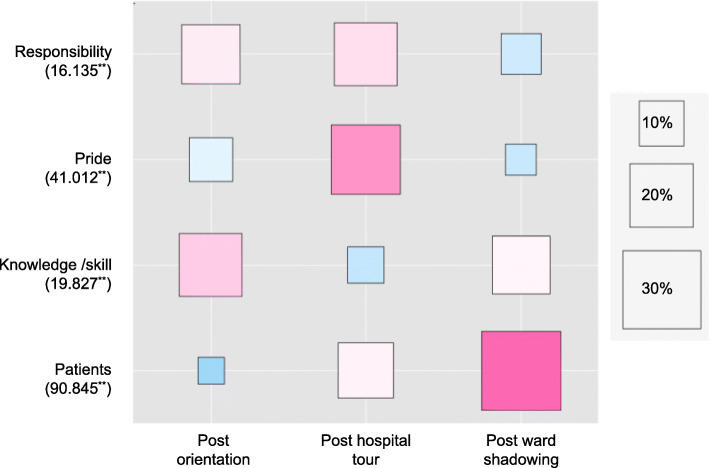
A comparison of the frequencies of the four most-commonly-used words describing professional characteristics across all three time points. The numbers in the brackets are *chi*-square values. ^**^p < 0.01

We found ‘medical care’ was almost always used with ‘patient’, for example, ‘best medical care for patients’ or ‘patient-centred medical care’. Here ‘patient’ was the most frequently mentioned term used in the statements. Figure 1 shows a comparison of the frequencies of the four most-commonly-used words (‘responsibility’, ‘pride’, ‘knowledge/skill’ and ‘patient’) across all three time points; the difference between frequencies at each point was significant (*p* < 0.01). Although students mainly focused on professional ‘knowledge/skills’ before the ECE placement, ‘pride’ and ‘responsibility’ were the most-commonly-used terms after the hospital tour. Additionally, ‘patient’, which was not present in the top 10% of extracted words before ECE, rose to the most-frequently-stated term after students completed the ward shadowing, but only to the 6th most-frequently-occurring after the hospital tour.

### Reflective essay key themes

**Fig. 2:**
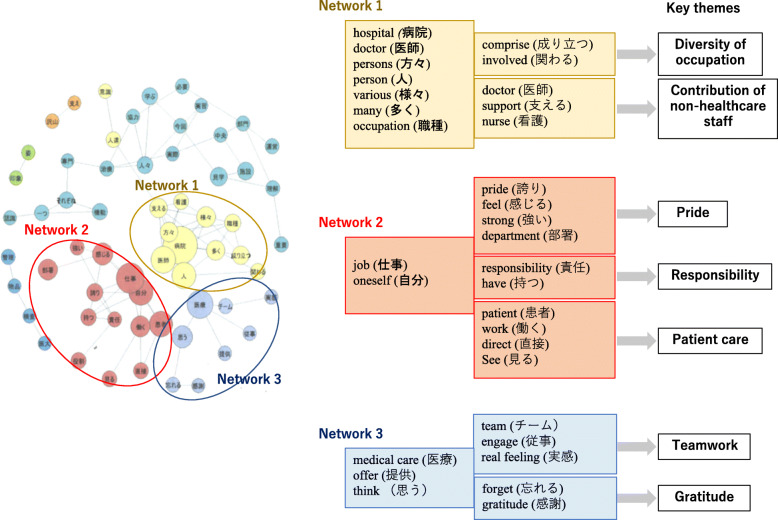
A breakdown of how the 50 most-commonly-occurring words extracted from the reflective essays distribute across the three emergent networks leading to the extracted themes

**Fig. 3:**
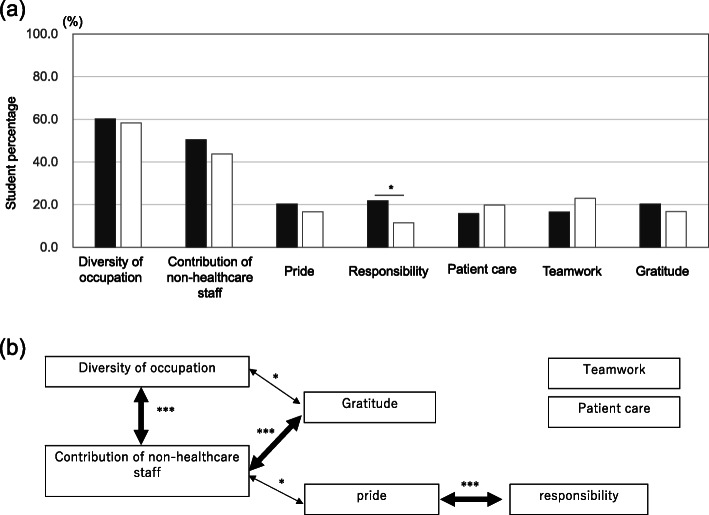
**a** Comparison of the frequencies of the seven extracted themes across the reflective essays, categorised by student self-reported impact. The black and white bars represent group A (most influenced by non-healthcare professionals) and group B (most influenced by clinical or allied health professionals), respectively. A chi- squared test was used to explore differences between the expression of themes depending on which department had the greatest self-reported impact on students. ^*^p < 0.05. **b** Diagrammatic representation of the significant interrelationships among the themes based on the results of Fisher’s exact test. ^*^p < 0.05, ^**^p < 0.01, ^***^p < 0.001

We proceeded to evaluate the impact of the hospital tour on students through text mining-aided thematic analysis of the 259 reflective essays submitted. KH coder detected a total of 1892 sentences, 3140 unique terms and through evaluation of the word interrelationships three distinct networks emerged. Figure 2 shows how the extracted words distribute across the networks and the organisation into seven themes. Examining the themes, ‘diversity of occupation’ was mentioned most often (59.9% of essays), closely followed by ‘contribution of non-healthcare professionals’ (48.7%), with 34.7% mentioning both. ‘pride’, ‘responsibility’, ‘teamwork’, ‘patient care’ and ‘gratitude’ were mentioned in 17.8, 16.6, 20.5, 18.5 and 18.9% of essays respectively. There was no difference between groups with respect to themes arising with the exception for ‘responsibility’ which came up more in those most affected by interactions with non-healthcare professionals (*P* < 0.05) (Fig. 3a).

### Contribution of non-healthcare professionals, diversity of occupation and gratitude

Students reported a change in their perception of the hospital team after encountering more job types during the modified ECE-placement than they initially described; we categorized this as ‘diversity of occupation’. Students emphasized the ‘contribution of non-healthcare professionals’ in supporting the activities of healthcare delivery and their integral role in hospital operations.

‘*Until now, I was only looking at doctors and nurses like a patient or visitor. I learnt how many occupations are involved in hospital management.’*

*‘I discovered [that] departments outside of medical care play important roles. Doctors are able to concentrate on their job thanks to those supporting the hospital behind the scenes.’*

We found that students who mentioned the ‘contribution of non-healthcare professionals’ more often referenced ‘diversity of occupation’ (*p* < 0.001), ‘gratitude’ (*p* < 0.001) and ‘pride’ (*p* < 0.05). Figure 3b shows the relationships between these terms, including how ‘contribution of non-healthcare professionals’, ‘diversity of occupation’ and ‘gratitude’ cluster together. Text analysis of these essays revealed that gratitude was expressed towards the non-healthcare professionals from a doctor’s perspective, recognising the role of non-healthcare professionals in supporting their future work.

*‘Every facility plays an important role, and if any of them disappeared, the hospital wouldn’t run smoothly. As a doctor, I felt I must pay respect to those working to support me.’*

### Pride and responsibility

Non-healthcare professionals showed enthusiasm in explaining their roles, prompting significant reflection in students irrespective of which professionals had the most self-reported influence on students.

*‘The [non-healthcare] staff explained their work so enthusiastically that I felt the pride they have in their work.’*

‘Pride’ and ‘responsibility’ were shown to be significantly connected (*p* < 0.001) (Fig. 3b). Students articulated the connection between ‘responsibility’ and ‘pride’ as the self-belief of non-healthcare professionals in their work, leading to embracement of responsibilities, resulting in pride of their part in an integrated whole.

*‘Every person had a sense of [their own] responsibility in supporting this hospital.’*

‘*Although they were not front stage, they were working like unsung heroes. The confidence generates a sense of responsibility which is a source of pride.’*

### Teamwork and patient care

Although ‘teamwork’ and ‘patient care’ were independent categories not statistically associated with other themes (Fig. 3b), the student perception of the medical care team expanded to include non-healthcare professionals during the placement. Students emphasized how all staff working in the hospital worked together for patients whether or not they had patient facing roles.

*‘[They said] “Even if I don’t see patients directly, my work leads to saving patients.” I felt everyone was working in this way.’*

*‘I realized that all staff cooperated as a team, because the main focus of their activity was patient care.’*

### Self-reported impact on students and the recognition of altruism

Of the departments visited during the placement, 51.4% of students stated those staffed by non-healthcare professionals had the greatest impact on them (group A), compared to 37.1% of students who found departments with medical and allied healthcare staff most influential (group B). Analysis of the essay texts showed students in groups A and B emphasized the strong sense of purpose demonstrated by non-healthcare professionals in their commitment to patients, awareness of responsibilities, and in their selfless attitude.

*‘Visiting the non-medical departments was even more interesting than visiting the medical ones. While the type of work was different, everyone was putting patients first.’*

*‘The words that stuck with me the most were, “Although I don’t expect you will ever meet anyone in this department again, you just need to know we are behind the scenes.” It was really cool.’*

Some students reflected more deeply on the selfless attitude displayed by non-healthcare professionals, elaborating on the extent of the dedication they witnessed, and concluded that the value lay in making the utmost effort for others at their own discretion without seeking reward.

## Discussion

We expanded the scope of our ECE programme to include socialization with non-healthcare professionals, applying text mining to reflective assignments to explore the impact on student professional identity. We found students positively responded to the altruism and dedication modelled by non-healthcare professionals and showed appreciation for their role in supporting healthcare delivery, leading to increased self-conceptualisation as a doctor.

Our results highlight how the majority of students were neither aware of the roles of less visible non-healthcare professionals nor how they contributed to healthcare provision prior to the placement. The experience deepened their understanding of the nature of being a doctor, from an individual supporting patients, to someone supported by a diverse ‘behind the scenes’ team with patient care the common goal. Furthermore, our results show how interaction with non-healthcare professionals initiated a shift in student perceptions of professional characteristics towards ‘pride [in one’s work]’ and ‘responsibility’, which was elaborated on in the reflective essays. This shift was not reproduced after ward-shadowing nor in the reflections of those most affected by interactions with healthcare professionals, suggesting that some attributes outside of patient care may be overshadowed in clinical settings. This supports findings that non-clinical interactions are important in developing medical professional identity [22].

Observing how other healthcare professionals interact with doctors is recognised as important in constructing physician professional identity [23], therefore it follows that perspectives from positions external to medical culture would be valuable. We feel the student descriptions of non-healthcare professionals fit within the paradigm of altruism. Altruism in medicine, formulated from the opposition to egoism [24], is defined as an overt voluntary behaviour with a beneficial outcome, where the executor makes a choice and incurs a cost that is not recuperated [25]. Altruism has been accepted as part of professional practice since the invocation of the Hippocratic oath [26], however there is debate whether the work of doctors is truly altruistic or part of fiduciary responsibility [24]. As the work of most non-healthcare professionals does not encompass responsibilities to patients in the same way as clinical work, their dedication irrespective of notice or thanks as perceived by the students, may represent a clearer version of altruism without the obfuscation imposed by the doctor-patient relationship. Such an interpretation strengthens our findings that non-healthcare professionals can have an active role in modelling prosocial attributes to medical students.

We found the most common theme reflected on by students was ‘diversity of occupation’, which led to student gratitude towards non-healthcare professionals for their role in supporting healthcare delivery. That students expressed gratitude from the perspective of doctors is an important outcome from our modified ECE placement. Kegan’s constructive developmental theory, adapted to professional identity formation in medicine and previously applied to Japanese students, underpins this [3, 23, 27]. We hypothesise that students taking on the perspective of doctors represent a transition from stage 2, where preconceived, narrow ideas of the characteristics and role of doctors prevail, to stage 3, where they are able to assume a more immersive identity, emulating the obligations and internalising the perspectives of doctors [3, 23]. In talking as doctors, students are negotiating their professional-self by articulating the discourse expected of the role [23]. Level 3 is one before that expected of a professional fully consolidated into their role ready to enter practice, and appropriate for our students’ current stage of training and a desirable outcome after ECE.

Culture and power are important lenses through which to consider our findings. Identities are created within power-knowledge paradigms, where individuals accept rules engendered through the application of regulatory power [28], therefore we might expect our students to be more strongly affected by the discourse of clinicians. Unexpectedly, we found the majority of students were more affected by the discourse of non-healthcare professionals. We hypothesise that due to a strong power differential between experts and novices, first-year students-with their relatively low level of medical knowledge, newcomer status and the short duration of the placement-may have felt closer to non-healthcare professionals, who co-exist with students as equals at the periphery of the medical ‘*community of practice*’ [2].

All our participants were Japanese, which imparts shared experiences in appreciating the contribution of others. Embedded in Japanese culture is inherent respect towards other professions, summarised by the proverb “職業に貴賤なし-*all professions are equal in honour*”. From an early age, Japanese school children are taught to clean their own classrooms and distribute meals to classmates, forming the grassroots of a society powered by shared responsibility and mutual respect [29, 30]. Additionally, there are influences from Bushido, the personal code of samurai warriors, which permeates modern social consciousness [31]. Bushido centres around seven virtues, two of which align with the western concept of altruism [32]. As a result, our students maybe more receptive to the contribution and prosocial attitudes of non-healthcare professionals.

Culture also shapes the values and behaviour of all Japanese professionals. Historically in Japan, work has been viewed as a privilege and a joy, and therefore something to be conducted with pride [33]. Elements of this remains to some degree today [33], particularly when there is strong sense of ownership and belief in organisational goals, which in this case is patient care. It is this cultural link between ‘*responsibility*’ and ‘*pride*’ that was reflected on and articulated by the students, showing the acquisition of local cultural norms through our placement. Social identity theory states that individuals seek to identify with groups that demonstrate traits most pertinent to their identity and self-esteem, and through positive interactions with others their own identity is affirmed [28]. The effect on both the non-healthcare professionals and students during our study can therefore be modelled as a transactional process of ‘*identity capital*’ [28], where through their mutual positive interaction, acceptance and recognition, both groups acquired stronger self-actualisation.

Based on our findings, the timing of the placement is an important consideration. We propose that the optimal time to socialise students with non-healthcare professionals is before they have fully consolidated their physician identity, to incorporate prosocial behaviours from other fields. Socialization of this nature represents an important step in internalising working cultural norms and learning to function as part of wider society [1, 23]. As such, ECE may offer a limited window to take advantage of this intersection between professional identity development and legitimate peripheral participation. This window could represent a unique opportunity for student appreciation of the place of medical practice within the heterogeneity of professional interactions in the wider hospital environment, however further studies are needed.

Although some of our findings have limited generalisability outside Japan, the underlying message of the inherent gains socialisation with non-healthcare professionals brings has global implications for ECE design. It also highlights future directions in research exploring the varied contributions of team members in the wider hospital environment (clinical and non-clinical), whose position, external to the physician role, may uniquely support professional identity development and lead to their increased recognition as role models for medical students. Whilst our research is rooted in Japanese culture, it shows the need for future studies within other distinct cultural contexts in order to support student acquisition of workplace norms, which are not explicitly taught but expected of all professionals entering the workplace. Our study is however subject to other limitations. The reflective assignments were compulsory with the intention of being evaluated by faculty, which might have influenced the content. Further issues arise with the utilisation of text-mining, as every word is counted as a separate instance, although we minimised this exaggeration effect through choosing a thematic approach in our analysis. Additionally, it was not possible to compare student experiences to those of students undertaking previous ECE placements, and a sequential effect cannot be ruled out arising from the order that students undertook the modified ECE placement. Further exploration of interaction with non-healthcare professionals during ECE is required, including into the long-term effects on professional identity development and the impact on hospital interactions as a practising doctor. Our results highlight an important role for non-healthcare professionals in the professional identity development of medical students. This aligns service-dominant logic in the organisation of health professional education, which prioritises co-productive partnerships with all stakeholders [34]. Involving non-healthcare professionals in the education of medical students represents a move towards this model, engaging all members of the community in educating future members.

## Conclusion

Socialisation with non-healthcare professionals during ECE promoted the recognition of altruism and responsibility, initiating a shift in student perceptions of professional characteristics and increased their self-conceptualisation as doctors. Non-healthcare professionals may have an active role in facilitating the acquisition of society’s cultural norms, supporting student integration and participation as members in the hospital community.

## Data Availability

The anonymised dataset used and/or analysed during the current study are available from the corresponding author on reasonable request.
